# Factors affecting risk perception of COVID-19: differences by age and gender

**DOI:** 10.3389/fpubh.2024.1484306

**Published:** 2025-01-22

**Authors:** Sungwook Kang, Chang Hoon You, Young Dae Kwon

**Affiliations:** ^1^Department of Health Services Management, Daegu Haany University, Gyeongsan, Republic of Korea; ^2^Department of Healthcare Policy, Seoul Public Health Research Institute, Seoul Medical Center, Seoul, Republic of Korea; ^3^Department of Humanities and Social Medicine, College of Medicine, The Catholic University of Korea, Seoul, Republic of Korea; ^4^Catholic Institute for Public Health and Healthcare Management, The Catholic University of Korea, Seoul, Republic of Korea

**Keywords:** COVID-19, risk perception, Korea, gender differences, age differences

## Abstract

**Introduction:**

Addressing emerging infectious diseases is a major task in public health. This study investigated the factors influencing the perception of risk related to COVID-19.

**Methods:**

This study analyzed data from the 2020 Social Survey conducted nationwide in South Korea, targeting 34,909 individuals aged 13 years and older. Using an ordered logit regression model, we examined the relationship between COVID-19 risk perception and its predictors across age groups (20–44 years, 45–64 years, 65 years and older) and gender groups. The predictors included in the analysis model were demographic factors, socioeconomic factors, health and quality of life factors, levels of social trust, and climate change factors.

**Results:**

The results demonstrated that COVID-19 risk perception was higher among older individuals and women compared with men. In the young population group (20–44 years), mental stress was related to COVID-19 risk perception, but this was not observed in other population groups. In the older adult population group (65 years and older), education level was related to COVID-19 risk perception, whereas this was not observed in the young population group. In the male group, economic variables such as income and employment status were related to COVID-19 risk perception, whereas in women, family-related variables such as marital status and housing type were related. In most subgroup analyses, lower income levels or lower life satisfaction were associated with higher COVID-19 risk perception.

**Discussion:**

The findings of this study suggest that health authorities need to tailor their responses to COVID-19 risk perception based on different populations and social groups. For the older adult population with a high-risk perception, it is necessary to provide reliable information to reduce anxiety caused by excessive risk perception. For the young population, proactive responses from health authorities regarding mental health are needed.

## Introduction

In recent times, several infectious diseases have been critical challenges to human health, such as Ebola virus disease, severe acute respiratory syndrome, Middle East respiratory syndrome, and coronavirus disease 2019 (COVID-19). The World Health Organization (WHO) identified emerging infectious diseases as the greatest threat to human health in the future (1). Therefore, identifying the best response to infectious diseases like COVID-19 has become a major public health challenge. The most important measure in a society-wide response to infectious diseases is to encourage individuals to engage in infection prevention behaviors to prevent the spread of infection. According to the health belief model, the degree to which individuals engage in health prevention behaviors depends on how seriously and sensitively they perceive future risks (2–4). Previous studies have shown that differences in risk perception led to variation in the implementation of COVID-19 infection prevention behaviors, such as hand washing and vaccination (5–7). Therefore, identifying the factors that influence the risk perception of COVID-19 can help develop effective strategies to respond to infectious diseases in the future.

After Chinese authorities reported the existence of the COVID-19 virus to WHO in January 2020, COVID-19 represented a serious threat for 3 years until WHO lifted the international public health emergency declaration in May 2023. As of February 4, 2024, the total number of confirmed cases of COVID-19 worldwide was 774,593,066, with a total of 7,028,881 deaths (8). The degree of infection and severity of COVID-19 showed significant differences by age. The number of deaths and fatality rates were significantly higher in the older adult population compared with other age groups. In South Korea, as of August 31, 2023, the proportion of the older adult population aged 60 years and over among the total confirmed cases of 34,572,554 was 20.8%, while the proportion of the older adult population among the total deaths of 35,605 was 93.8% (9). These differences in mortality rates by age group seem to have led to differences in the risk perception of COVID-19 by age group. Previous studies showed that the older adult population has a higher risk perception of the severity of COVID-19 (6, 7, 10–12). In contrast, other studies suggest that age is not related to the perception of COVID-19 infection (13, 14) or that the risk perception even decreases with age (15–17).

Population cohorts share unique socio-cultural perceptions, which can differentiate them from other cohorts. South Korea is a country that has experienced rapid socio-economic changes since the 1950s. After the Korean War that lasted 3 years, Korea was one of the poorest countries in 1953, but as of 2021, it has grown into an economic powerhouse with a per capita GDP of $32,422 (18) and the world’s 13th largest economy (19). Despite a long period of military dictatorship from 1961 to 1987, Korea achieved democracy through the efforts of civil society. Because of these rapid social changes in Korean society, different population cohorts have distinctive socio-cultural perceptions (20). These differences in perception may also lead to differences in risk perception of COVID-19.

Generally, women are reported to be more risk-averse than men, which leads to a higher tendency for women to purchase insurance and engage more actively in disease prevention behaviors (21–24). Women also showed higher risk perceptions of COVID-19 infection compared with men (25, 26). Despite the higher COVID-19 fatality rate among men, the risk perception of men is lower than that of women (27), and even when information about risks specific to men is provided to them, men’s risk perception does not tend to increase (28). In addition to age and gender, various socio-demographic variables have been studied as risk factors for COVID-19. Differences in COVID-19 risk perception were observed based on marital status (11, 29, 30), household composition (31), educational attainment (13, 32–35), and income level (35, 36). Furthermore, health status (32, 37) and quality of life (38) were also associated with COVID-19 risk perception.

Perceived risk is influenced by factors such as knowledge about the risk, voluntariness of exposure to the risk, visibility of the risk, and social trust (39, 40). Given that COVID-19 is a novel disease, transmitted through a virus that cannot be seen, and faced with low initial public trust in many countries’ healthcare systems and authorities, it was anticipated that the perceived risk of COVID-19 would be very high. Previous studies have shown that the perceived risk of COVID-19 varies depending on the information and knowledge about the virus and the level of trust in health authorities, medical institutions, and healthcare professionals (13, 32, 41). On the other hand, the ecological crisis is one of the most significant perceived risks globally, with climate change being the most visible aspect. Both COVID-19 and climate change share similarities in that they have global impacts that are not confined to a single country or specific areas of society and stem from ecological imbalances (42–46). For this reason, climate change can be assumed as a predictive factor for COVID-19 risk perception.

The COVID-19 pandemic provided an experimental environment to examine how different social and demographic groups perceive risk. While risk situations faced by different social and demographic groups typically differ, the COVID-19 pandemic placed everyone in the same risk situation simultaneously. This situation provided a useful environment for comparing and analyzing how risk perception differs among social and demographic groups. This study examined differences in the predictive factors of COVID-19 risk perception by age cohort (young adults, middle-aged, older adult) and between men and women.

## Methods

### Data and subjects

This study used the survey data from the 2020 Social Survey conducted by Statistics Korea. The Statistics Korea Social Survey was conducted to investigate social interests and subjective awareness related to the quality of life of the population, to understand the level of living and social changes, and to provide basic data for social development policies. The Social Survey has been conducted since 2008 with the purpose of collecting basic data for the development of social indicators. The survey is divided into 10 areas: welfare, social participation, culture and leisure, income and consumption, labor, health, education, safety, family, and environment. The survey cycle involves splitting these 10 areas into two groups, with five areas each, and conducting the survey every 2 years.

The 2020 Social Survey collected data on five areas, in addition to basic items: family, education and training, health, crime and safety, and living environment. The survey targeted 42,281 household members aged 13 years and older living in approximately 19,000 sample households nationwide. The survey period was from May 13 to May 28, 2020, spanning 16 days. In the current study, from the 42,281 respondents in the 2020 Social Survey, we excluded 7,323 respondents under the age of 20 and 49 respondents who did not respond to the risk perception of emerging infectious diseases, resulting in a final analysis sample of 34,909 respondents.

### Variables and measurements

In this study, the dependent variable selected was the ‘perceived risk level of emerging infectious diseases’ surveyed in the Social Survey. This variable was measured using a 5-point scale (very safe, safe, neutral, unsafe, and very unsafe). Several variables were included as predictors for the perceived risk of COVID-19. The predictor variables in this study consisted of demographic factors, socioeconomic factors, health and quality of life factors, social trust levels, and climate change factors.

Demographic factors consist of gender, age, marital status, household composition, presence of school-aged children, and residential areas. Age was categorized into 20–44 years, 45–64 years, and 65 years and older. Marital status was classified as unmarried (including separated, divorced, widowed, or never married) and married. Household composition was divided into single-person households and others. Presence of school-aged children was categorized as living with school-aged children and others. Residential areas were categorized into the metropolitan area (Seoul, Incheon, and Gyeonggi Province) and other areas.

Socioeconomic factors consist of education, income, and economic activity. Education level was divided into middle school or less, high school graduate, and college or higher. Income level was categorized based on the monthly household income: less than two million Korean won (KRW), 2–5 million KRW, 5–8 million KRW, and more than 8 million KRW. Economic activity was determined based on whether the individual worked for at least 1 h for income in the past week.

Health and quality of life factors consist of health status, stress, regular sleep, life satisfaction, and satisfaction with the living environment. Health status was measured based on illness during the past 2 weeks. Stress was measured on a 4-point scale (highly, moderate, slightly, and not at all) based on the level of stress experienced in general daily life over the past 2 weeks. Regular sleep was measured by whether the individual slept 6–8 h a day. Life satisfaction and satisfaction with living environment (air, water, soil, and noise) were measured on a 5-point scale (excellent, good, even, bad, and worst).

Social trust levels and climate change factors consist of trust in others’ law abidance, the willingness to share costs for environmental pollution, and anxiety about climate change. Trust in others’ law abidance was measured by how well the respondent thinks others follow the law on a 5-point scale (extremely, highly, moderate, slightly, and not at all). Willingness to share costs for environmental protection was measured on a 5-point scale (strongly agree, agree, neutral, disagree, and strongly disagree). Anxiety about climate change was measured by how anxious the respondent felt about climate change on a 5-point scale (extremely, highly, moderate, slightly, and not at all).

### Statistical analysis

The differences in perceived COVID-19 risk based on predictor variables were analyzed using univariate analysis. To examine the relationship between the predictor variables of perceived COVID-19 risk and perceived COVID-19 risk itself, a multivariate analysis was conducted using an ordered logit regression model. Furthermore, to verify the relationship between the predictor variables and perceived COVID-19 risk for different age groups (20–44 years, 45–64 years, 65 years, and older) and between male and female groups, multivariate analysis was conducted using ordered logit regression models for each subgroup. Statistical analysis was performed using STATA 14.2 (StataCorp LP, College Station, TX, United States). The alpha level of 0.05 (two-tailed) was considered the threshold for statistical significance.

## Results

Among the study participants, 52.3% were women and the age distribution was as follows: 39.6% were 45–64 years old, 34.5% were 20–44 years old, and 25.6% were 65 years or older. In terms of education level, 44.2% had some college education or higher. Additionally, 63.7% were married, and 58.0% were currently engaged in economic activities. Regarding health and quality of life, 33.6% of participants had been ill in the past 2 weeks, and 12.7% were dissatisfied with their life. In the overall group, 10.1% believed that others do not comply well with the law, and 45.2% had a high level of risk perception regarding climate change (Table 1).

**Table 1:** Characteristics of study participants and levels of COVID-19 risk perception (*n* = 34,909).

Characteristics	Frequency	%	Risk perception for COVID-19 (Mean ± SD)	Chi/F	*p*
Gender				197.3	<0.001
Male	16,661	47.7	3.49 ± 1.13		
Female	18,248	52.3	3.66 ± 1.11		
Age (years)				713.0	<0.001
20–44	12,052	34.5	3.37 ± 1.16		
45–64	13,917	39.9	3.57 ± 1.10		
≥65	8,940	25.6	3.86 ± 1.04		
Education				501.2	<0.001
≥College	9,075	26.0	3.89 ± 1.03		
High school	10,409	29.8	3.64 ± 1.09		
≥College	15,425	44.2	3.35 ± 1.01		
Marital status				1.22	0.223
Married	22,234	63.7	3.57 ± 1.11		
Unmarried	12,675	36.3	3.58 ± 1.13		
Presence of school-aged children				24.1	<0.001
Yes	8,221	23.6	3.31 ± 1.13		
No	26,688	76.5	3.66 ± 1.11		
Metropolitan area				7.7	<0.001
Metropolitan	9,641	27.6	3.60 ± 1.12		
Others	25,268	72.4	3.50 ± 1.14		
Household composition				10.2	<0.001
Single person	5,838	16.7	3.71 ± 1.10		
Others	29,071	83.3	3.55 ± 1.12		
Income (unit: 10 K KRW)				227.5	<0.001
<200	7,665	41.5	3.56 ± 1.12		
200–499	7,779	42.1	3.81 ± 1.06		
500–799	2,270	12.3	3.26 ± 1.13		
≥800	775	4.2	3.17 ± 1.12		
Economic activity				9.6	<0.001
Yes	20,236	58.0	3.53 ± 1.13		
No	14,673	42.0	3.64 ± 1.12		
Illness during the past 2 weeks				23.3	<0.001
Yes	11,712	33.6	3.77 ± 1.09		
No	23,197	66.5	3.48 ± 1.13		
Daily stress during the past 2 weeks			40.52	369.8	<0.001
Highly	1,821	5.2	3.78 ± 1.14		
Moderate	15,456	44.3	3.61 ± 1.10		
Slightly	14,608	41.9	3.53 ± 1.11		
Not at all	3,024	8.7	3.47 ± 1.25		
Regular sleep				6.39	<0.001
Yes	28,313	81.1	3.56 ± 1.12		
No	6,596	18.9	3.65 ± 1.12		
Life satisfaction^a^				327.4	<0.001
Excellent/good	13,988	40.1	3.41 ± 1.17		
Even	16,469	47.2	3.64 ± 1.07		
Bad/worst	4,452	12.7	3.86 ± 1.07		
Satisfaction with living environment^a^				177.8	<0.001
Excellent/good	17,039	48.8	3.48 ± 1.18		
Even	14,961	42.9	3.63 ± 1.04		
Bad/worst	2,909	8.3	3.86 ± 1.07		
Trust in others’ law abidance^a^				387.5	<0.001
Extremely/highly	17,792	51.0	3.43 ± 1.17		
Moderate	13,584	38.9	3.67 ± 1.04		
Slightly/not at all	3,533	10.1	3.95 ± 1.07		
Willingness to share costs for environmental pollution^a^				319.4	<0.001
Strongly agree/agree	17,748	50.8	3.45 ± 1.18		
Neutral	11,986	34.4	3.62 ± 1.03		
Disagree/strongly disagree	5,175	14.8	3.89 ± 1.07		
Anxiety about climate change^a^				110.6	<0.001
Extremely/highly	15,794	45.2	3.66 ± 1.14		
Moderate	11,058	31.7	3.56 ± 1.04		
Slightly/not at all	8,057	23.1	3.44 ± 1.18		
Total	34,909	100.0	3.58 ± 1.26		

a Although the variable was measured on a 5-point scale, it was reclassified into a 3-point scale for analysis purposes.

KRW, Korean won; SD, standard deviation.

In terms of demographic factors related to COVID-19 risk perception, women had a higher risk perception of COVID-19 (OR = 1.224, *p* < 0.001). Risk perception increased with age (45–64 years: OR = 1.158, *p* < 0.001; ≥65 years: OR = 1.321, *p* < 0.001). Lower education levels were associated with a higher risk perception (high school: OR = 1.158, *p* < 0.001; ≥college: OR = 1.321, *p* < 0.001). Married individuals had a higher risk perception (OR = 1.130, *p* < 0.001). Those without school-aged children had a higher risk perception (OR = 0.697, *p* < 0.001). Single-person households had a higher risk perception (OR = 1.106, *p* < 0.001). The interaction effect of gender and age showed that, compared with women, men had a higher increase in risk perception with age (male & ≥65 years OR = 1.162, *p* < 0.001). Regarding economic predictors to COVID-19 risk perception, lower income levels were associated with a higher risk perception (2–5 million KRW: OR = 0.920, *p* = 0.004; 5–8 million KRW: OR = 0.789, *p* < 0.001; ≥8 million KRW: OR = 0.745, *p* < 0.001). Current economic activity was associated with a higher risk perception (OR = 1.091, *p* < 0.001).

Individuals in poorer health had a higher risk perception than those in good health (OR = 1.143, *p* < 0.001). Lower life satisfaction was associated with a higher risk perception (fair: OR = 1.117, *p* < 0.001; poor/worst: OR = 1.355, *p* < 0.001). Those who perceived worsening living environments had a higher risk perception (fair: OR = 1.164, *p* < 0.001; bad/worst: OR = 1.599, *p* < 0.001). Those who thought others did not comply with the law had a higher risk perception (moderate: OR = 1.287, *p* < 0.001; slightly/not at all: OR = 1.956, *p* < 0.001). Higher risk perception of climate change was associated with higher COVID-19 risk perception (moderate: OR = 1.114, *p* < 0.001; extremely/highly: OR = 1.445, *p* < 0.001). Those unwilling to bear the cost of environmental pollution had a higher risk perception (neutral: OR = 1.149, *p* < 0.001; disagree/strongly disagree: OR = 1.702, *p* < 0.001) (Table 2).

**Table 2:** Analysis of the relationship between COVID-19 risk perception and its predictors.

Variable	Category	ORs	*P*
Gender	Male (ref.)		
	Female	1.224	<0.001
Age (year)	20–44 (ref.)		
	45–64	1.158	<0.001
	≥65	1.321	<0.001
Gender × age (year)	Male & 20–44 (ref.)		
	Male & 45–64	1.032	0.493
	Male & ≥65	1.162	<0.001
Education	≤Middle school (ref.)		
	High school	0.867	<0.001
	≥College	0.661	<0.001
Marital status	Unmarried (ref.)		
	Married	1.130	<0.001
Presence of school-aged children	No (ref.)		
	Yes	0.697	<0.001
Metropolitan area	Others (ref.)		
	Metropolitan	0.888	<0.001
Household composition	Others (ref.)		
	Single person	1.106	0.002
Income (unit:10 K KRW)	≺200 (ref.)		
	200–499	0.920	0.004
	500–799	0.789	<0.001
	≥800	0.745	<0.001
Economic activity	No (ref.)		
	Yes	1.091	<0.001
Illness during the past 2 weeks	No (ref.)		
	Yes	1.143	<0.001
Daily stress during the past 2 weeks	Not at all (ref.)		
	Slightly	1.166	0.016
	Moderate	1.094	0.036
	Highly	1.069	0.101
Regular sleep	No (ref.)		
	Yes	0.937	0.011
Life satisfaction^a^	Excellent/good (ref.)		
	Even	1.117	<0.001
	Bad/worst	1.355	<0.001
Satisfaction with living environment^a^	Excellent/good (ref.)		
	Even	1.164	<0.001
	Bad/worst	1.599	<0.001
Trust in others’ law abidance^a^	Extremely/highly (ref.)		
	Moderate	1.287	<0.001
	Slightly/not at all	1.956	<0.001
Willingness to share costs for environmental pollution^a^	Strongly agree/agree (ref.)		
	Neutral	1.149	<0.001
	Disagree/strongly disagree	1.702	<0.001
Anxiety about climate change^a^	Slightly/not at all (ref.)		
	Moderate	1.114	<0.001
	Extremely/highly	1.445	<0.001

a Although the variable was measured on a 5-point scale, it was reclassified into a 3-point scale for analysis purposes.

OR, odds ratio; KRW, Korean won; ref, reference.

Table 3 shows the results from the analysis of the relationship between COVID-19 risk perception and its predictors by age group. In the young adult population, young women had a higher level of COVID-19 risk perception compared with young men (OR = 1.153, *p* < 0.001). In the group with school-aged children (OR = 0.842, *p* < 0.001), those living in the metropolitan area (OR = 0.894, *p* = 0.002), and those with higher income levels (2–5 million KRW, OR = 0.841, *p* = 0.001; 5–8 million KRW, OR = 0.811, *p* < 0.007; ≥8 million KRW, OR = 0.729, *p* = 0.010), the level of COVID-19 risk perception was lower. In the group that had been ill in the past 2 weeks (OR = 0.894, *p* = 0.043), the level of COVID-19 risk perception was lower. However, frequent daily stress (slightly, OR = 1.639, *p* < 0.001; moderate, OR = 1.501, *p* < 0.001; highly, OR = 1.450, *p* < 0.001) was associated with a higher the level of COVID-19 risk perception. In the middle-aged population, daily life stress factors and regular sleep patterns were not associated with COVID-19 risk perception. However, all other variables showed a significant association.

**Table 3:** Analysis of the relationship between COVID-19 risk perception and its predictors by age group.

Variable	Category	20–44 year		45–64 year		≥65 year	
		ORs	*p*	ORs	*p*	ORs	*p*
Gender	Male (ref.)						
	Female	1.158	<0.001	1.247	<0.001	1.097	0.048
Age	Year	0.9999	0.987	1.027	<0.001	1.001	0.642
Education	≤Middle school (ref.)						
	High school	0.960	0.778	0.830	<0.001	0.888	0.026
	≥College	0.785	0.085	0.613	<0.001	0.788	0.001
Marital status	Unmarried (ref.)						
	Married	1.055	0.237	1.182	<0.001	1.204	0.004
Presence of school-aged children	No (ref.)						
	Yes	0.842	<0.001	0.639	<0.001	0.595	0.087
Metropolitan area	Others (ref.)						
	Metropolitan	0.894	0.002	0.854	<0.001	0.993	0.894
Household composition	Others (ref.)						
	Single person	0.965	0.530	1.152	0.013	1.294	<0.001
Income (unit:10 K KRW)	≺200 (ref.)						
	200–499	0.841	0.001	0.997	0.961	0.982	0.778
	500–799	0.811	0.007	0.848	0.008	00.67	0.008
	≥800	0.729	0.010	0.827	0.036	0.636	0.149
Economic activity	No (ref.)						
	Yes	1.035	0.350	1.090	0.016	1.180	<0.001
Illness during the past 2 weeks	No (ref.)						
	Yes	0.896	0.043	1.173	<0.001	1.285	<0.001
Daily stress during the past 2 weeks	Not at all (ref.)						
	Slightly	1.639	<0.001	1.084	0.433	1.016	0.895
	Moderate	1.501	<0.001	1.018	0.804	0.992	0.919
	Highly	1.450	<0.001	1.012	0.869	0.940	0.369
Regular sleep	No (ref.)						
	Yes	0.932	0.093	0.943	0.149	0.978	0.670
Life satisfaction^a^	Excellent/good (ref.)						
	Even	1.110	0.005	1.126	0.001	1.066	0.179
	Bad/worst	1.389	<0.001	1.415	<0.001	1.231	0.004
Satisfaction with living environment^a^	Excellent/good (ref.)						
	Even	1.395	<0.001	1.124	<0.001	0.976	0.575
	Bad/worst	1.689	<0.001	1.757	<0.001	1.308	0.002
Trust in others’ law abidance^a^	Extremely/highly (ref.)						
	Moderate	1.321	<0.001	1.378	<0.001	1.113	0.010
	Slightly/not at all	1.679	<0.001	2.417	<0.001	1.750	<0.001
Willingness to share costs for environmental pollution^a^	Strongly agree/agree (ref.)						
	Neutral	1.214	<0.001	1.189	<0.001	1.007	0.858
	Disagree/strongly disagree	1.874	<0.001	1.778	<0.001	1.420	<0.001
Anxiety about climate change^a^	Slightly/not at all (ref.)						
	Moderate	1.239	<0.001	1.037	0.413	1.091	0.087
	Extremely/highly	1.404	<0.001	1.358	<0.001	1.735	<0.001

a Although the variable was measured on a 5-point scale, it was reclassified into a 3-point scale for analysis purposes.

OR, odds ratio; KRW, Korean won; ref, reference.

In the older adult population, factors such as gender (OR = 1.097, *p* = 0.048), education level (high school: OR = 0.888, *p* = 0.026; ≥college: OR = 0.788, *p* = 0.001), marital status (OR = 1.204, *p* = 0.008), single-person household (OR = 1.294, *p* < 0.001), economic activity (OR = 1.180, *p* < 0.001), and presence of illness (OR = 1.285, *p* < 0.001) were related to COVID-19 risk perception. However, age, stress, and regular sleep factors did not show significant relationships with COVID-19 risk perception. In all three groups, variables such as life satisfaction, satisfaction with living environment, and level of social trust were related to the perception of COVID-19 risk. However, the climate change variable did not show consistent results with the perception of COVID-19 risk in the older adult population group (Table 3).

Table 4 shows the results from the analysis of the relationship between COVID-19 risk perception and its predictors by gender. In both men and women, most socio-demographic variables, such as age and education, were associated with COVID-19 risk perception. In the male group, income level (2–5 million KRW, OR = 0.879, *p* = 0.001; 5–8 million KRW, OR = 0.729, *p* < 0.001; ≥8 million KRW, OR = 0.705, *p* < 0.001) and participation in economic activities (OR = 0.147, *p* < 0.001) were associated with COVID-19 risk perception. However, in the female group, neither of these variables showed an association with COVID-19 risk perception. Being ill in the past two weeks showed a positive association with COVID-19 risk perception in both male (OR = 0.118, *p* < 0.001) and female groups (OR = 0.155, *p* < 0.001). In the male group, higher daily life stress was associated with an increased risk perception (slightly: OR = 1.474, *p* < 0.001; moderate: OR = 1.265, *p* < 0.001; highly: OR = 1.202, *p* = 0.002), while in the female group, there was no significant association. Conversely, regular sleep was positively associated with COVID-19 risk perception in the female group (OR = 0.904, *p* = 0.004), but this was not significant in the male group (Table 4).

**Table 4:** Analysis of the relationship between COVID-19 risk perception and its predictors by gender group.

Variable	Category	Male		Female	
		ORs	*p*	ORs	*p*
Age (year)	20–44 (ref.)				
	45–64	1.202	<0.001	1.141	<0.001
	≥65	1.565	<0.001	1.254	<0.001
Education	≤Middle school (ref.)				
	High school	0.852	<0.001	0.890	0.007
	≥College	0.675	<0.001	0.653	<0.001
Marital status	Unmarried (ref.)				
	Married	1.154	0.002	1.132	0.001
Presence of school-aged children	No (ref.)				
	Yes	0.702	<0.001	0.690	<0.001
Metropolitan area	Others (ref.)				
	Metropolitan	0.846	<0.001	0.929	0.018
Household composition	No (ref.)				
	Single person	1.004	0.917	1.210	<0.001
Income (unit:10 K KRW)	≺200 (ref.)				
	200–499	0.879	0.001	0.922	0.138
	500–799	0.729	<0.001	0.903	0.364
	≥800	0.705	<0.001	0.728	0.087
Economic activity	No (ref.)				
	Yes	1.147	<0.001	1.052	0.075
Illness during the past 2 weeks	No (ref.)				
	Yes	1.118	0.001	1.155	<0.001
Daily stress during the past 2 weeks	Not at all (ref.)				
	Slightly	1.474	<0.001	0.957	0.615
	Moderate	1.265	<0.001	0.950	0.403
	Highly	1.202	0.002	0.950	0.382
Regular sleep	No (ref.)				
	Yes	0.986	0.714	0.904	0.004
Life satisfaction^a^	Excellent/good (ref.)				
	Even	1.124	<0.001	1.108	0.001
	Bad/worst	1.368	<0.001	1.341	<0.001
Satisfaction with living environment^a^	Excellent/good (ref.)				
	Even	1.127	<0.001	1.196	<0.001
	Bad/worst	1.539	<0.001	1.656	<0.001
Trust in others’ law abidance^a^	Extremely/highly (ref.)				
	Moderate	1.316	<0.001	1.262	<0.001
	Slightly/not at all	2.052	<0.001	1.874	<0.001
Willingness to share costs for environmental pollution^a^	Strongly agree/agree (ref.)				
	Neutral	1.167	<0.001	1.131	<0.001
	Disagree/strongly disagree	1.730	<0.001	1.674	<0.001
Anxiety about climate change^a^	Slightly/not at all (ref.)				
	Moderate	1.153	<0.001	1.072	0.071
	Extremely/highly	1.458	<0.001	1.427	<0.001

a Although the variable was measured on a 5-point scale, it was reclassified into a 3-point scale for analysis purposes.

OR, odds ratio; KRW, Korean won; ref, reference.

## Discussion

This study explored the factors related to risk perception of COVID-19 during the pandemic and further analyzed these factors by age and gender group. Analysis of the entire population group showed that most of the variables predicted in this study were related to COVID-19 risk perception, though there were some differences in subgroup analyses. For the young adult population, factors such as age, marital status, single-person household, and economic activity were not related to COVID-19 risk perception, while for the older adult population, factors like age, residence in the metropolitan area, and income did not show a relationship. For the middle-aged group, most of the predictive variables were similar to the overall population group in their relationship to COVID-19 risk perception. Economic factors were related to COVID-19 risk perception in males but not in females.

The results of this study indicate significant differences in COVID-19 risk perception across age cohorts in Korea. Since January 2020, when the first COVID-19 cases were reported in Korea, up until May 2020 when the survey was conducted, there were nearly no deaths due to COVID-19 among the young adult group (20–44 years). In contrast, there was a high incidence and mortality rate among the older adult (9). The global pandemic situation and the fact that COVID-19 is more fatal to the older adult likely caused the differences in risk perception across age groups. The higher the level of social trust in health authorities and the more knowledge and information there is about COVID-19, the lower the perceived risk of COVID-19 (39, 40). Considering that COVID-19 is a novel infectious disease and the global pandemic situation, health authorities should promptly provide reliable information about COVID-19 to prevent the older adult population from falling into unnecessary risk perception. On the other hand, there was no significant relationship between age and COVID-19 risk perception in both the very low-risk group (20–44 years) and very high-risk group (≥65 years), aligning with previous studies that found no association between age and COVID-19 risk perception (13, 14, 41). This indicates that including age as a continuous variable in single-year increments has limitations meaningfully explaining changes in the perception of COVID-19 risk.

Studies have shown that women worry more about the future and are less likely to take risks compared with men (27, 47). Research has reported weak correlations between gender and risk perception (48). Women have been found to have a higher risk perception of COVID-19 compared with men (13, 25–28, 41). The results of this study confirm that women generally have a higher risk perception of COVID-19 compared with men. Among various risks, women are known to have a higher perception of health-related risks compared to men (49, 50). Additionally, women are reported to perceive involuntary risks more strongly than men (51). Therefore, it is considered that women have a higher risk perception of COVID-19, an involuntary health threat, compared to men. In examining gender differences, women showed a higher correlation between family-related factors such as marital status and household composition with COVID-19 risk perception. In contrast, men correlated more with economic factors (monthly income, economic activity). These results reflect the characteristics of men being more economically active and women having stronger emotional ties through family. When examining the interaction of age and gender, the middle-aged group did not show significant gender interactions compared with the young adult group, but the older adult group did. This indicates that gender differences in risk perception are more pronounced in the older adult population. Older women’s general health status and socio-economic position are lower than older men (52), which may explain the more significant gender differences in risk perception among the older adult compared with other age groups. These results suggest that health policy authorities should consider providing targeted policy attention to older women within the senior population.

Higher education levels were associated with lower risk perception for both the middle-aged (45–64 years) and older adult (≥65 years) group. This supports the hypothesis that higher levels of knowledge about risks lead to easier access to information about those risks, thereby lowering risk perception (27, 28, 33). Higher education levels increase understanding and information about emerging infectious diseases like COVID-19, which can reduce anxiety and risk perception about the disease. In contrast, the young adult group (20–44 years) did not show significant differences in risk perception by education level, consistent with previous studies suggesting no significant relationship between education level and risk perception of COVID-19 (13, 32, 34, 53). The education level of the young adults in Korea is generally very high; as of 2020, 98.6% of the young adults (20–44 years) had at least a high school diploma. This high level of education likely minimizes the difference in COVID-19 information across educational levels. Additionally, young adults have high access to information via the internet, so there may be no significant difference in knowledge about COVID-19 based on educational level. These findings suggest that health authorities should adopt differentiated approaches for different age groups. Specifically, education and information about COVID-19 should be tailored to middle-aged and older populations with relatively lower levels of education.

This study found that single-person households in the middle-aged group had a higher risk perception compared with non-single-person households. This result can be interpreted as single-person households experiencing a higher risk perception due to the importance of in-house isolation and treatment during COVID-19. The South Korean government implemented strict mobility control policies between February 5 and early May 2020, when the survey in this study was conducted. These measures included travel bans, workplace closures, and school closures. As a result, it can be inferred that individuals living alone developed a heightened perception of risk regarding COVID-19. In the young adult population (20–44 years), the effect of single-person household on risk perception was minimal, given the generally low risk perception in this group. It is known that individuals living alone tend to have poorer mental health compared to those who do not live alone (54–56). Notably, older adults living alone are reported to experience higher rates of depression than younger individuals living alone (57, 58). During the pandemic, when mobility was strictly restricted, a distinct perception of risk regarding COVID-19 was evident among older adult individuals living alone. These findings suggest that health authorities should provide more targeted and thoughtful policy support for socially vulnerable groups, such as older adult individuals living alone.

Individuals engaged in economic activities had higher risk perception than those not engaged, indicating that stronger government regulations, such as travel bans and workplace closures to curb the spread of COVID-19, led those involved in economic activities to perceive more risk. Conversely, lower income levels were associated with higher risk perception, as individuals with lower income tend to have less job security and are more sensitive to economic downturns caused by COVID-19. South Korea implemented some of the most stringent mobility restrictions during the early stages of the COVID-19 pandemic, earning recognition as a leading country in pandemic control (59). However, these strict measures also caused a downturn in the domestic market, and the resulting economic slowdown is believed to have heightened the perception of COVID-19 risk among economically vulnerable populations.

Previous studies have reported a strong association between risk perception of COVID-19 and health (32, 37), and this study also found that individuals who had been ill in the past 2 weeks had a higher risk perception. Given that those with underlying health conditions have higher mortality rates from COVID-19 (60, 61), it is understandable that those in poor health would perceive higher risks from COVID-19. However, this association might also reflect reverse causality, where high risk perception could negatively impact physical and mental health. Overall, in the young population with high health levels, no association was found between the prevalence of illness in the past 2 weeks and risk perception. Instead, in the young population, higher levels of stress were associated with higher levels of risk perception. Previous research has shown that COVID-19 negatively impacts mental health, such as increasing depression and stress (62–64). This negative impact is likely more pronounced among young adults, who experience significant disruptions to their active social lives due to COVID-19, and this stress may contribute to a higher risk perception. These results underline the need for health authorities to pay special attention to the mental health of younger populations made vulnerable by COVID-19.

Risk perception is influenced by emotional factors such as satisfaction (65). This study shows that lower satisfaction with a subjective quality of life is associated with higher risk perception of COVID-19, demonstrating a link between emotions and risk perception. This suggests the need for appropriate education and information for socioeconomically marginalized groups with a low quality of life to reduce an unnecessary risk perception related to new infectious diseases like COVID-19.

Studies have shown that higher levels of trust in society are associated with lower perceptions of risk (13, 39, 40). In this study, trust in others’ law abidance was established as a proxy variable for the level of social trust. This study found a negative relationship between the level of trust in society and risk perception. This indicates that trust in health authorities during the COVID-19 pandemic might be able to reduce excessive anxiety about the virus. The study found that higher awareness of the climate crisis and higher support for environmental burden-sharing were associated with higher COVID-19 risk perception. This suggests that a more significant concern about climate change and a stronger sense of social responsibility regarding environmental issues are associated with a higher risk perception of COVID-19. Climate change is not a problem confined to a single nation but a global-level threat, and the fact that its causes stem from ecological imbalances makes it similar to the COVID-19 pandemic. This resemblance enhances the perceived connection between the two variables (44–46). Among different population groups, the young adult group showed a high association between social trust and climate change variables with COVID-19 risk perception, while the older adult group showed less association. This indicates that the young adult group recognizes the connection between the climate crisis and COVID-19 more than the older adult group. These results reflect that younger generations are more concerned about future sustainability issues, such as climate change and ecological crises, than older generations (66, 67).

There are several limitations to this study. First, the variables measuring COVID-19 risk perception were not diverse. Another limitation is the inability to assess the appropriateness of the level of COVID-19 risk perception, particularly whether the high-risk perception among the older adult is exaggerated. A third limitation is that the survey did not include questions about COVID-19 preventive behaviors, so the relationship between COVID-19 risk perception and preventive behaviors could not be examined. The final limitation is that although related studies suggest that quarantine measures during the COVID-19 pandemic may have influenced risk perception (68, 69), this variable could not be included in the analysis due to limitations in the data used in this study.

However, a key strength of this study is its analysis of data from a survey of the entire national population shortly after the declaration of the COVID-19 pandemic. The strength of the data used in this study lies in its broad inclusion of various characteristics, such as the demographic and socioeconomic traits of the respondents, their health and quality of life, social trust, and perceptions of climate change. In the early stages of the COVID-19 pandemic, previous studies on risk perception were conducted across the populations of many countries worldwide. However, research papers focusing on the perception of risk among Koreans are rare, and this paper is considered the first of its kind. COVID-19 is not a disease confined to a single nation but a novel infectious disease of a global scale that has profoundly impacted all of humanity. For this reason, comparative studies between countries are highly significant in COVID-19 research, and this study is expected to provide essential foundational data and significant implications for such comparative research.

## Conclusion

The COVID-19 pandemic is an external, independent challenge affecting all generations and genders equally. This study confirms that there are differences in COVID-19 risk perception by age and gender groups and that there are differences in risk factors. The results provide meaningful basic data for health authorities needing risk management and policy tailored to different socio-demographic groups. For the older adult population with a relatively high-risk perception, providing reliable information about emerging infectious diseases is essential to reduce unnecessary anxiety. The strong association between mental stress and COVID-19 risk perception in the young adult group indicates the need for proactive measures by health authorities to address mental health issues in this group. Socioeconomically disadvantaged individuals with a lower income or life satisfaction also show a higher risk perception, and health policies should focus on reducing excessive risk perception among these vulnerable groups.

## Data Availability

Publicly available datasets were analyzed in this study. This data can be found at: https://www.kostat.go.kr/board.es?mid=a20111050000&bid=11761&act=view&list_no=386681.
